# Generation of synthetic tympanic membrane images: Development, human validation, and clinical implications of synthetic data

**DOI:** 10.1371/journal.pdig.0000202

**Published:** 2023-02-24

**Authors:** Krish Suresh, Michael S. Cohen, Christopher J. Hartnick, Ryan A. Bartholomew, Daniel J. Lee, Matthew G. Crowson

**Affiliations:** 1 Department of Otolaryngology-Head & Neck Surgery, Massachusetts Eye & Ear, Boston, Massachusetts, United States of America; 2 Department of Otolaryngology-Head & Neck Surgery, Harvard Medical School, Boston, Massachusetts, United States of America; Emory University, UNITED STATES

## Abstract

Synthetic clinical images could augment real medical image datasets, a novel approach in otolaryngology–head and neck surgery (OHNS). Our objective was to develop a generative adversarial network (GAN) for tympanic membrane images and to validate the quality of synthetic images with human reviewers. Our model was developed using a state-of-the-art GAN architecture, StyleGAN2-ADA. The network was trained on intraoperative high-definition (HD) endoscopic images of tympanic membranes collected from pediatric patients undergoing myringotomy with possible tympanostomy tube placement. A human validation survey was administered to a cohort of OHNS and pediatrics trainees at our institution. The primary measure of model quality was the Frechet Inception Distance (FID), a metric comparing the distribution of generated images with the distribution of real images. The measures used for human reviewer validation were the sensitivity, specificity, and area under the curve (AUC) for humans’ ability to discern synthetic from real images. Our dataset comprised 202 images. The best GAN was trained at 512x512 image resolution with a FID of 47.0. The progression of images through training showed stepwise “learning” of the anatomic features of a tympanic membrane. The validation survey was taken by 65 persons who reviewed 925 images. Human reviewers demonstrated a sensitivity of 66%, specificity of 73%, and AUC of 0.69 for the detection of synthetic images. In summary, we successfully developed a GAN to produce synthetic tympanic membrane images and validated this with human reviewers. These images could be used to bolster real datasets with various pathologies and develop more robust deep learning models such as those used for diagnostic predictions from otoscopic images. However, caution should be exercised with the use of synthetic data given issues regarding data diversity and performance validation. Any model trained using synthetic data will require robust external validation to ensure validity and generalizability.

## Author summary

Synthetic clinical images could augment real medical image datasets with diverse and rare pathologies. Such synthetic data would have applications in medical education and in bolstering datasets to improve the performance of machine learning models such as diagnostic classifiers. Our study represents one of the first generative models for synthetic image data within our field of Otolaryngology–Head and Neck Surgery. We use a state-of-the-art generative adversarial network (GAN) architecture on a limited dataset to develop a model that produces photo-realistic synthetic images of the tympanic membrane. We developed this model using a small training set of just 202 images. The successful production of photo-realistic synthetic images using a small training set demonstrates the potential for synthetic data approaches with limited datasets, as is often the case in clinical medicine. We validate our images with human reviewers to determine how well humans can distinguish real from synthetic images. This human Turing Test validation approach is rare in synthetic data studies and provides unique insights into the quality of generated images. Future studies could use synthetic tympanic membrane images to train improved diagnostic classifiers based on otoscopic images. However, caution must be exercised with the use of synthetic data, and robust external validation of models trained using synthetic data will be necessary.

## Introduction

Deep learning has seen numerous successful applications in medicine including classification of histopathologic images, prediction of clinical outcomes from the electronic health record, and phenotype prediction from the human genome [1]. Within otolaryngology–head and neck surgery, deep learning has been applied to numerous domains ranging from hearing aid optimization to prediction of hearing outcomes from imaging data [2]. Machine learning for the diagnosis of ear infections is a particularly active area of research and commercial enthusiasm [3–7].

Generative adversarial networks (GANs) are a type of deep learning algorithm that generate synthetic data [8]. GANs have promising applications in biomedicine to overcome data scarcity and homogeneity, by generating large quantities of diverse data [9]. Within medicine, GANs have been used to generate images of skin lesions [10], H&E histopathology [11], colonic mucosa [12], COVID-19 chest X-rays [13], and more complex radiologic images [14,15]. This synthetic data is particularly valuable when it can compensate for situations where natural data is limited, such as rare or orphan diseases and situations where acquisition of additional data is not feasible.

Here, we report the development of a GAN for tympanic membrane (TM) image synthesis. We provide a summary of model development and illustrate progression of the generated images through training as the network learned various features of a TM. We also report the results of a validation test, where humans were asked to classify TM images as fake or real to assess the degree to which synthetic images could be differentiated from their real counterparts. We discuss the practical implications of synthetic data, urging caution and the need for robust validation. If properly applied, synthetic data in otolaryngology–head and neck surgery may expand the scope of deep learning in the field by providing large quantities of data for analysis and experimentation, including but not limited to the many rare pathologies encountered.

## Methods

This study protocol was approved under the Mass General Brigham Institutional Review Board protocol number 2019P003086.

### Data source

Intraoperative images of tympanic membranes were collected from pediatric patients undergoing myringotomy with possible tympanostomy tube placement for recurrent acute otitis media or otitis media with effusion between November 2019 and September 2020. Consent was exempted under the IRB listed above. Inclusion criteria for the images were greater than 75% visibility of the tympanic membrane, sufficient image quality for distinguishing major anatomic landmarks (i.e., annular ligament, malleus umbo), and normal appearance of the tympanic membrane without middle ear fluid as ascertained by myringotomy. Images were taken using a 0-degree 2.7 mm Hopkins rod telescope coupled to a high-definition (HD) camera (Karl Storz SE & Co KG, Tuttlingen, Germany), which captured images at 1920 x 1080 pixel resolution.

### GAN development

The GAN in this work was developed using the StyleGAN2-ADA architecture [16]. StyleGAN2-ADA was chosen because it was the state-of-the-art GAN architecture at the time this project was undertaken, and it was felt to be highly appropriate for this work given the introduction of adaptive discriminator augmentation permitting GAN development with limited datasets. Since that time, further approaches for image generation have gained traction (i.e. diffusion models), and these merit further exploration in future studies.

Raw images were adapted to train networks at 3 different resolutions: 256x256, 512x512, and 1024x1024. The dataset was amplified twofold through x-axis inversion, whereby an image and its mirror were both included in the training set.

GAN quality was assessed every 200 kimg (1 kimg = 1000 images shown to the discriminator) by the Frechet inception distance (FID) [16]. FID compares the distribution of generated images with the distribution of real images. A lower FID value is better and indicates more similar distributions between the real and synthetic image sets. GAN training was carried out as long as the FID continued to decrease, until an inflexion point was reached and FID started to increase. This indicated network convergence, the point at which the discriminator no longer provides meaningful feedback to the generator. At this point, the generator network image output quality collapses and training is stopped. For the final network, we additionally calculated precision and recall. In the context of GANs, precision is a measure of image fidelity, or the proportion of the generated image distribution that falls within the real image distribution. Recall is a measure of image diversity, or the proportion of the real image distribution that falls within the generated image distribution. Model training was performed on Google Compute Engine, provisioning a virtual machine with 8 NVIDIA V100 GPUs.

### Human validation survey

Human reviewers were tasked with assessing the photo-realism of the synthetic tympanic membrane images produced by the GAN. Principally, we assessed humans’ ability to detect a GAN-generated fake versus a real image of a tympanic membrane. The survey contained 15 tympanic membrane images, 8 fake and 7 real. These numbers were chosen to obtain a reasonable number of responses per reviewer while ideally reducing survey fatigue to promote survey completion, allowing for a fully crossed design. Each survey was scored as the number of correct responses divided by the number of total responses (partial responses were included). Narrative feedback was optionally solicited at the conclusion of the survey. The survey was distributed to a convenience sample of otolaryngology and pediatrics residents at our institution. Results were analyzed to assess humans’ ability to correctly classify the images as fake or real, calculating sensitivity, specificity, and area under the receiver operating characteristic curve (AUC). Inter-rater reliability was calculated for completed surveys using Light’s Kappa. Analysis was carried out in R (R Foundation for Statistical Computing, Vienna, Austria) on RStudio (RStudio Inc., Boston, MA).

## Results

**Fig 1:**
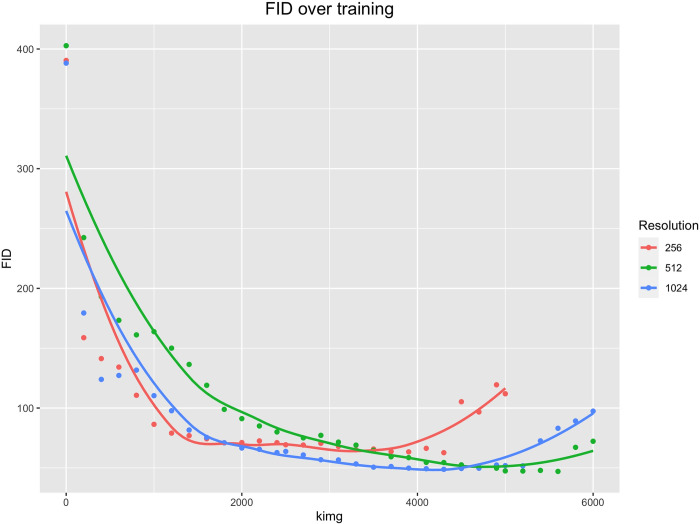
Frechet inception distance (FID) over the course of training for each resolution. 1 kimg = 1,000 images shown to the discriminator.

Our model training dataset comprised 202 endoscopic normal tympanic membrane images, which were amplified to 404 images through x-axis inversion data augmentation. **Fig 1** shows the FIDs across the training process for each of the three image resolutions for which networks were trained. For 256 image resolution, training was stopped at 5000 kimg as the network demonstrated clear signs of convergence after 4200 kimg. For 512 and 1024 resolutions, training was continued to 6000 kimg as the network continued to improve until 5600 kimg (512 resolution) and 5200 kimg (1024 resolution). The network with the best FID was chosen for further study–this was the 512 resolution network at 5600 kimg, with a FID of 47.0. The precision and recall of the final network were 66.4% and 9.4% respectively. The training time for the final model (512 resolution over 6000 kimg) was 15.80 hours.

**Fig 2:**
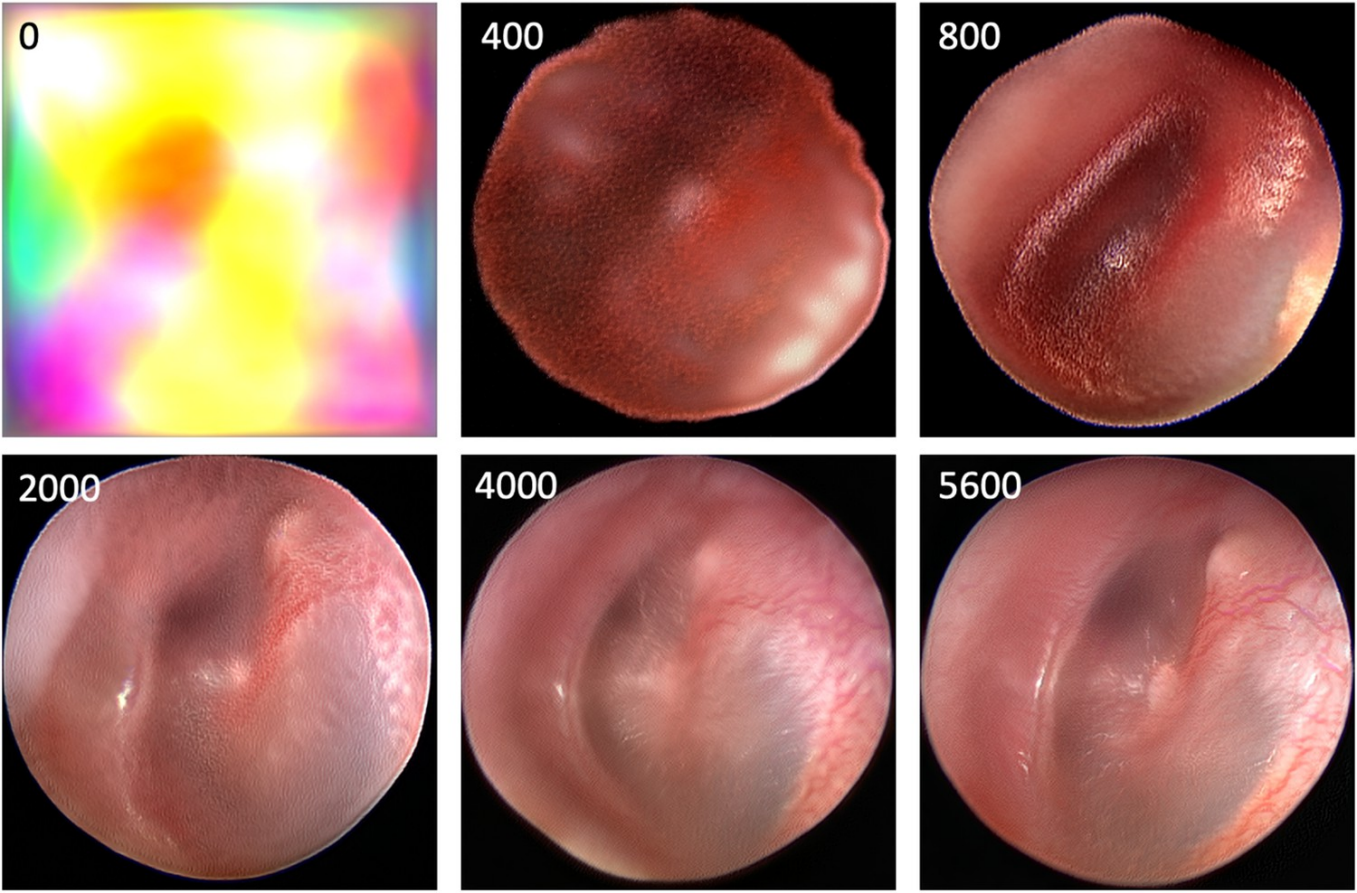
Progression of generated images and learned tympanic membrane features through training. The number in the top left corner of each image is the # kimg of the 512 network at which the image was generated.

**Fig 2** depicts the progression of the generated images across training. At the outset the generator is producing random noise (FID 402). At 400 kimg (FID 193), the network has learned the shape of an endoscopic image, and the shadow of the Eustachian tube and the light reflex begin to emerge. At 800 kimg (FID 161), the annulus is appreciable, and the malleus begins to take shape. At 2000 kimg (FID 91), the image quality is improved such that a tympanic membrane is recognizable, and the malleus and its lateral process are visible. At 4000 kimg (FID 54), image quality is further improved and the vascular strip takes form. At 5600 kimg (FID 47), the final network, there is a realistic image of a tympanic membrane, and notably the quality of the vasculature is further improved.

**Fig 3:**
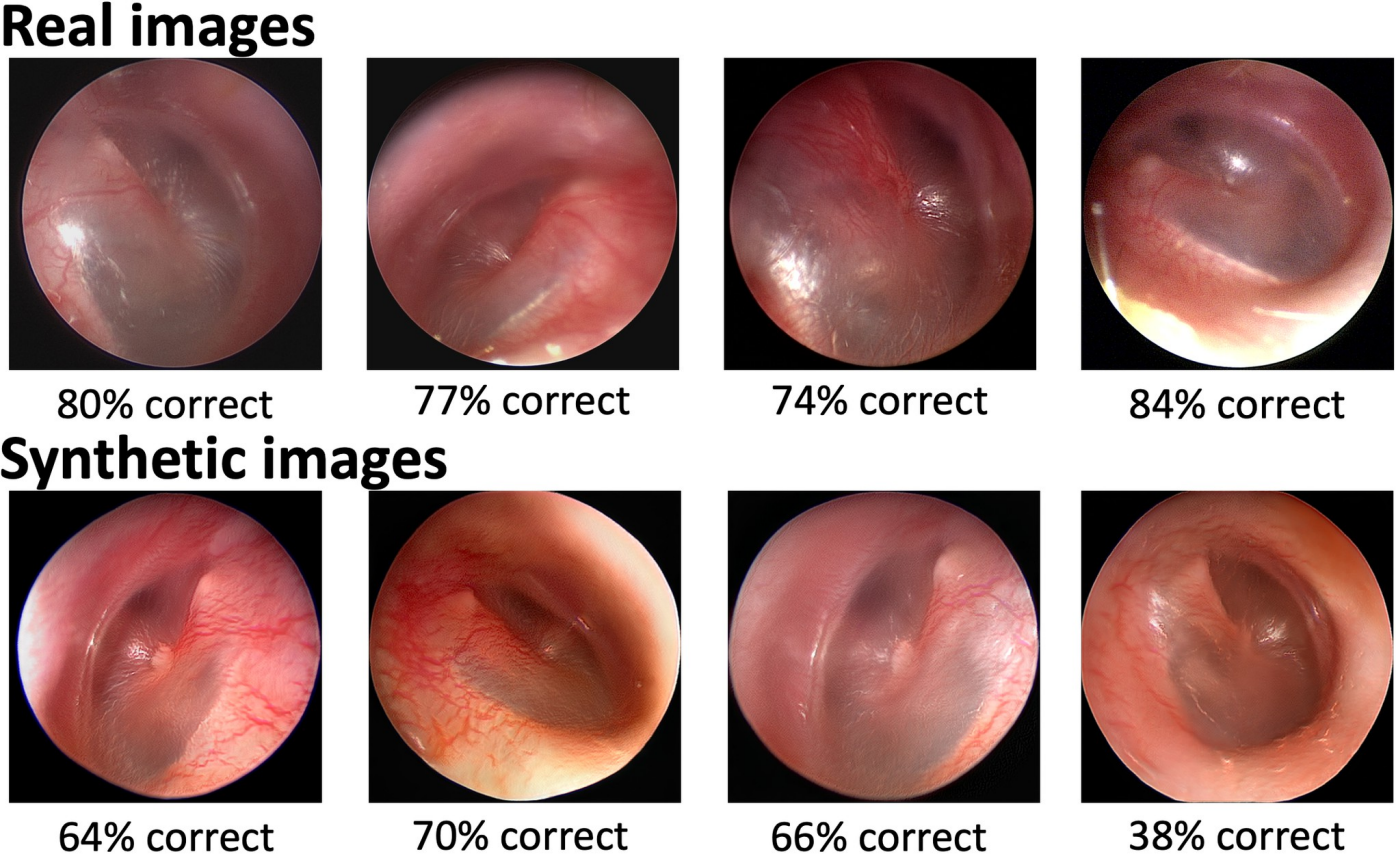
Sample survey images. A representative sample of 4 real (top row) and 4 synthetic (bottom row) survey images is shown. The number below each image indicates the percentage of responses that correctly classified the image as real or synthetic. For example, for the top left image, 80% of responses correctly classified this as a real image. For the bottom left image, 64% of responses correctly classified this as a synthetic image.

The human validation survey was distributed to 103 clinician trainees and taken by 65 for an aggregate survey response rate of 63% (otolaryngology-head & neck surgery n = 19 [29%], pediatrics n = 46 [71%]). A representative sample of 4 real and 4 synthetic survey images is shown in **Fig 3**, along with the percentage of responses that correctly classified each image as real or synthetic. In total, there were 925 individual image human responses. The average score was 70% in correctly determining the nature of the presented image (“real” or “fake”). Stratified by specialty, the mean score was 74% for otolaryngology and 68% for pediatrics (p = 0.27). A contingency table of human classification of fake and real images is shown in **Table 1**. The sensitivity of humans for detecting fake images was 66% and the specificity was 73%. The AUC was 0.69 (95% CI = 0.66–0.24). 60/65 surveys were completed; inter-rater reliability was 0.176. A common theme from the narrative feedback solicited at the end of the survey was that several respondents (n = 7; 11%) felt that the appearance of the vasculature “gave away” the fakes. Other comments made note of blurriness and strange light quality in the fake images.

**Table 1:** Contingency table of human image classification.

	Fake Image	Real Image
**Classified as Fake**	325	117
**Classified as Real**	168	315

## Discussion

In this work, we report the development of a generative adversarial network for synthetic tympanic membrane images and validate this through a human Turing test. We developed models across three different image resolutions and found the best model to be at 512 resolution after training for 5600 kimg with a Frechet inception distance (FID) of 47.0. While our network generated realistic, high-fidelity images of the human tympanic membrane (precision 66.4%), generated image diversity was low (recall 9.4%). Studying the progression of a generated image through model development provides important insights into how the generative adversarial network learns to delineate anatomically accurate aspects of a tympanic membrane.

Our human reviewer validation study provided additional insight into the performance of our GAN. We hypothesized that human reviewers would be able to differentiate between ‘real’ and ‘synthetic’ tympanic membrane images. Overall, humans had a sensitivity of 66% for detecting fake images and a specificity of 73% with an AUC of 0.69. These results suggest that humans performed better than random chance, however in many instances could not reliably discriminate between real and fake images–the ideal case with an effective generative adversarial network. Furthermore, the inter-rater reliability was 0.176, indicating poor agreement between respondents on which images were fake and which were real. Several respondents remarked that appearance of the vasculature ‘tipped’ them off that a given image was fake. Reviewing these images retrospectively, this could be because the vasculature constitutes one of the most detailed, finely pixelated parts of the images, so any noise around the vessels may have been more noticeable.

As noted above, generated image fidelity was high, however generated image diversity was low. The reasons for poor image diversity are unclear and this is an active area of research in synthetic data generation; this may in part be related to training with a limited dataset. It is notable that our GAN was able to generate realistic TM images by training on just 202 real images–this has significant implications in medicine, where limited datasets are the norm and hamper model development. Previously, GANs have required substantial volumes of data to train, on the scale of 10^5^–10^6^ images [16]. A recent technical development called adaptive discriminator augmentation (ADA) has enabled the development of GANs with far smaller datasets. The authors of the ADA algorithm reported data on GANs with small datasets that serves as a useful reference for our data: they developed a human faces GAN using 1000 images and achieved a FID of 21; without ADA, the same GAN was only able to achieve a FID of 100 [16]. The corresponding recall for this network is not available, however for the next step up, the network trained with 2000 images using ADA had a recall of 13.5%. For our GAN, the best resolution-based FID of 47.0 is promising considering our limited training dataset of 202 images. Our low recall of 9.4% is in line with other networks trained with limited data. Overall, the ability to train GANs with limited datasets could expand the applications of GANs in medicine, however image diversity is lacking.

The applications of GANs in medicine are in their infancy; significant enthusiasm is building, however this must be tempered by concerns regarding the use and validation of synthetic data. As is proposed in our work, a key application of GANs is to bolster real datasets, especially with rare diseases and/or underrepresented populations. In our use case, a GAN could be used to generate large quantities of photorealistic synthetic images of tympanic membranes, including rare pathologies. This data could in turn be used to train other tympanic membrane deep learning models, such as a diagnostic classifier that makes predictions from otoscopic images–this is currently a very active area of research and commercial enthusiasm [3–6]. Our group has previously published on the development of one such model for the diagnosis of pediatric middle ear effusion, [7] and it is conceivable that the model’s performance could be improved with the addition of thousands of synthetic images. This approach is not without precedent–a similar approach has been reported for the classification of renal cell carcinoma histology subtypes [9]. A model was trained using 10,000 histopathologic images, then, using the same dataset, a GAN was developed to generate an additional 10,000 images to bolster the original dataset including the rare chromophobe subtype. The diagnostic model trained with the real and synthetic data performed better than the model trained with the real data alone [9]. Another group reported similar outcomes for a GAN trained to synthesize computed tomography (CT) images of liver lesions [17].

While synthetic data offers an attractive way to generate vast quantities of diverse data, there are methodological issues that must be considered. First, there is no consensus on the best way to validate synthetic data. A human Turing test as used in our work is prone to inter-observer and intra-observer variabilities. Additionally, human tests are not scalable, and they may not be adaptable to more complex types of data. Other options include quantitative metrics such as FID, precision, and recall, however such metrics can be difficult to interpret and furthermore may “not reflect specific failure modes in the generation of synthetic data.” [9] Further research is needed on how best to validate synthetic data. Second, it is unclear whether it would be appropriate to use synthetic data to train other deep learning models, for example classification models. There would be concerns regarding the quality of the data above, and also regarding the diversity of generated images. Image diversity, also known as mode coverage, is a known problem for GANs. Alternative frameworks such as diffusion models have been shown to have superior mode coverage and warrant further exploration, especially given the requirement for diverse images in training classification models [18]. Given these issues, if synthetic data is used in the development of other machine learning models, it would be critical to validate those models on external datasets. This is true of any machine learning model, including those trained with only real data; however external validation would be even more important with synthetic data given concerns regarding the validity of the data itself as above [19]. If the models are proven externally valid, then they could begin to be used cautiously.

The limitations of our work are as noted above, regarding the inherent issues surrounding synthetic data. Another important limitation is that currently, we are restricted to generation of normal TM images. To develop a model that generates pathologic TM images will require a similar quantity of data to train, and acquisition of pathologic data is slower than the acquisition of more abundant normal data. This work is currently in progress and will be essential to maximize the utility of synthetic data, for example to train classification models, which requires normal and abnormal data. One final limitation is that while we were able to generate photo-realistic synthetic images with a relatively small training set of real images, subsequent network development with larger training sets is likely to improve synthetic image fidelity.

## Conclusion

Synthetic data has the potential to address major issues of data scarcity and homogeneity in biomedical research [9]. The development and validation of a tympanic membrane GAN as described in our work could lead to a library of plentiful and diverse images of tympanic membranes–this data could be useful in the development of diagnostic models for otoscopic images and other tools [7]. However, we urge caution with the use of synthetic data, given open questions regarding validation as well as the need for robust external validation of models trained with synthetic data.
